# Macrophage Heterogeneity in the Immunopathogenesis of Tuberculosis

**DOI:** 10.3389/fmicb.2018.01028

**Published:** 2018-05-23

**Authors:** Mohlopheni J. Marakalala, Fernando O. Martinez, Annette Plüddemann, Siamon Gordon

**Affiliations:** ^1^Division of Immunology, Department of Pathology, Institute of Infectious Disease and Molecular Medicine, University of Cape Town, Cape Town, South Africa; ^2^Faculty of Health and Medical Sciences, University of Surrey, Guildford, United Kingdom; ^3^Botnar Research Centre, NDORMS, University of Oxford, Oxford, United Kingdom; ^4^Nuffield Department of Primary Care Health Sciences, University of Oxford, Oxford, United Kingdom; ^5^Graduate Institute of Biomedical Sciences, College of Medicine, Chang Gung University, Taoyuan City, Taiwan; ^6^Sir William Dunn School of Pathology, University of Oxford, Oxford, United Kingdom

**Keywords:** macrophage, immunopathogenesis, tuberculosis, pulmonary, macrophage heterogeneity4, *Mycobacterium tuberculosis*

## Abstract

Macrophages play a central role in tuberculosis, as the site of primary infection, inducers and effectors of inflammation, innate and adaptive immunity, as well as mediators of tissue destruction and repair. Early descriptions by pathologists have emphasized their morphological heterogeneity in granulomas, followed by delineation of T lymphocyte-dependent activation of anti-mycobacterial resistance. More recently, powerful genetic and molecular tools have become available to describe macrophage cellular properties and their role in host-pathogen interactions. In this review we discuss aspects of macrophage heterogeneity relevant to the pathogenesis of tuberculosis and, conversely, lessons that can be learnt from mycobacterial infection, with regard to the immunobiological functions of macrophages in homeostasis and disease.

## Introduction

Tuberculosis (TB), caused by the bacterial pathogen *Mycobacterium tuberculosis* (MTB), is an airborne infection that primarily affects the lungs, and remains a major global health problem responsible for 1.5 million deaths annually (WHO)^1^. Upon inhalation, MTB organisms seed the alveolar space in the lung, where they are captured by alveolar macrophages. This is followed by an early inflammatory response, induced by both bacterial and host factors, which results in recruitment of leukocytes from neighboring blood vessels to the site of infection. These include monocytes which differentiate into various populations of tissue macrophages, such as epithelioid cells, foamy macrophages and multinucleated Langhans giant cells, to create macrophage-rich granulomatous lesions (Taylor et al., 2006; Volkman et al., 2010). Initially, granulomas are cellular aggregates of the different macrophages and other innate immune cells surrounded by fibroblasts (Ulrichs et al., 2004; Ehlers and Schaible, 2012; Guirado and Schlesinger, 2013). With the onset of adaptive immunity, granulomas acquire a more solid, intact structure with a lymphocytic cuff composed of T and B cells at their periphery. Later in infection, granulomas can undergo complex remodeling. For reasons not yet fully understood, the solid granuloma can accumulate necrotic damage that results in the formation of caseum at the center. Necrotic granulomas may undergo liquefaction to form cavitary lesions, giving the bacteria access to nearby airways and thus the ability to spread (Russell et al., 2010) within the lung and elsewhere within the body.

Granulomas have been assigned wide-ranging functions, some of which also depend on the animal model studied. In zebrafish, for example, granulomas have been depicted as vehicles that help spread the pathogen to other sites within the organism (Ramakrishnan, 2012). Different investigators have described granulomas as a successful method of sequestering a chronic pathogen (Saunders and Cooper, 2000; Nathan, 2016). However, if containment fails, granulomas may simply become structures that provide nutrients and a niche for the bacteria to replicate without immune restriction (Peyron et al., 2008).

Macrophages are the predominant host cells implicated in entry, growth and restriction within the infected host. Both MTB and macrophages are heterogeneous in metabolic activity and their interaction determines the outcome of infection. Before considering various aspects of the host-pathogen response to infection, we summarize present understanding of the heterogeneous origin, differentiation and activation of monocytes and tissue macrophages, the dispersed organ known as the Mononuclear Phagocyte System (MPS).

## Heterogeneity of macrophages and pathogens

There has been a paradigm shift in our understanding of the origin, differentiation, distribution and properties of the cells of the MPS (Ginhoux and Guilliams, 2016; Gordon and Plüddemann, 2017). Almost all current knowledge is based on studies in the mouse, and, although not identical in detail, the broad outlines are likely to be similar in the human. The main shift in our thinking arises from discovery of the mixed origins of mouse macrophages from yolk sac and fetal liver precursors during development, and postnatally from bone marrow. Embryo-derived “monocytes” and macrophages seed tissue resident macrophages throughout the body, turning over slowly at local sites where they persist throughout adult life. As required, e.g., in the gut (Bujko et al., 2018), tissue-resident macrophages are replenished from bone marrow hematopoietic stem cells (HSC), to a variable extent in different organs.

**Figure 1:**
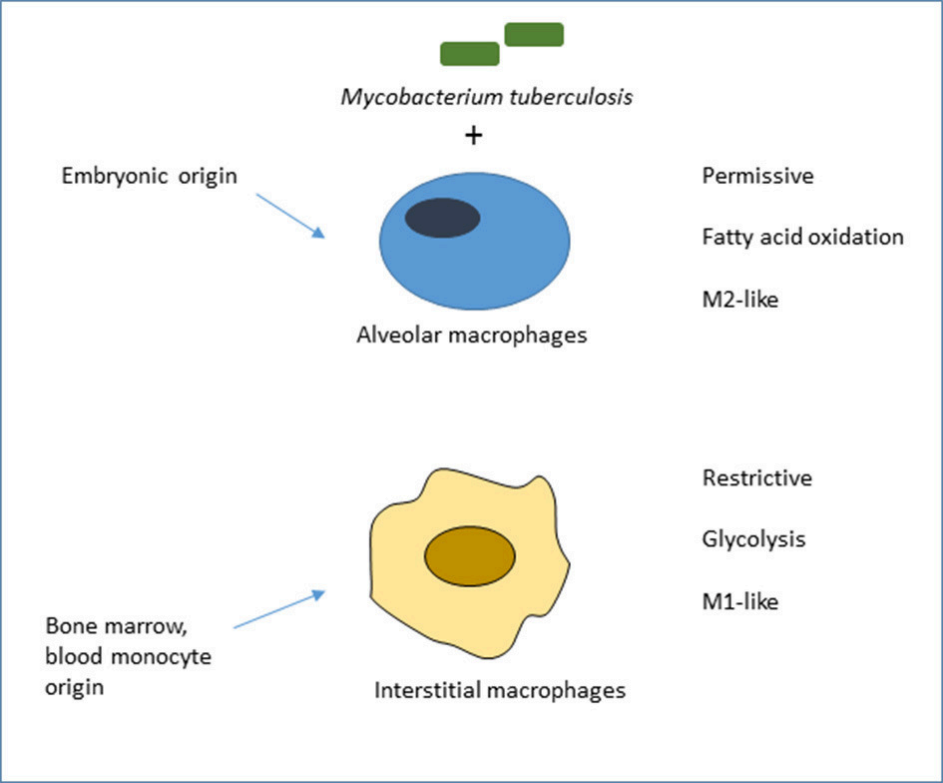
Ontogeny of mouse lung macrophages shapes the outcome of initial MTB infection *in vivo*. Adapted from Huang et al. (2018), who compared the role of alveolar macrophages (AM), of embryonic origin, with mainly monocytic, bone marrow-derived interstitial macrophages (IM) in acute infection of mice by fluorescent MTB reporter strains. Bacilli in AM, deriving energy from fatty acid oxidation, grew more readily than IM, which were actively glycolytic, more restrictive and expressed M1-like rather than M2-like markers of macrophage activation. The authors favored a model of pre-programming, linked to ontogeny, rather than adaptation to the distinct local microenvironment. There is suggestive, but no direct, evidence in humans that alveolar and other tissue-resident macrophages are of embryonic origin. This conclusion is based on transplantation studies of donor and recipient macrophages in skin (Bigley et al., 2011) and small intestine (Bujko et al., 2018), and evidence of local turnover in lung rather than bone marrow origin. It is not known which plasma membrane receptors mediate uptake of MTB in this *in vivo* model. Alveolar macrophages in the mouse express low levels of F4/80 and of CD11b, and high levels of several plasma membrane receptors: lectins such as the mannose receptor (CD206), and the Scavenger Receptor AI/II and MARCO, which are involved in the uptake of inhaled particles such as MTB (Gordon et al., 2014a). Other widely studied lectins expressed by lung macrophages include the FcRgamma-coupled receptors Dectin-2, Mincle and MCL, as well as Dectin-1, Mannose Receptor and DC-SIGN (Marakalala and Ndlovu, 2017).

Bone marrow-derived monocytes are themselves heterogeneous within the circulation, where they can remain, enter tissues constitutively or be recruited in response to a variety of sterile or infectious stimuli. They are loosely described as inflammatory, elicited or recruited monocyte-derived macrophages, and are known to display markedly different properties to those of tissue-resident macrophages. In response to poorly understood local environmental stimuli, e.g., in the lung, liver, gut, brain and skin, these macrophage populations adopt different tissue-specific phenotypes (Lavin et al., 2014), which can influence the outcome of MTB infection. As described below and shown schematically in Figure 1, Russell and colleagues have used a pulmonary infection model in the mouse to establish for the first time, the importance of the distinction between embryonic and bone marrow origin of lung macrophages in determining the outcome of initial infection by MTB (Huang et al., 2018).

Recent population and single cell analysis of macrophages, *in situ* and *ex vivo*, illustrate the extensive heterogeneity of plasma membrane receptor (Gordon and Plüddemann, 2017) and biosynthetic gene expression (Lavin et al., 2014), of macrophages in the steady state and in various intracellular infection models. In addition, macrophages in different organs express distinct tissue-specific phenotypes, as well as canonical genes shared by macrophages in different tissues (e.g., Lavin et al., 2014). Intriguingly, phagocytosis of apoptotic cells downregulates inflammatory responses irrespective of tissue macrophage location (reviewed in Gordon and Plüddemann, 2018). Conversely, Avraham and colleagues have shown that pathogen cell-to-cell variability drives Type 1 Interferon production by individual macrophages in a Salmonella model; this was ascribed to PhoPQ activity in the population of invading bacteria that modified LPS in a subset of organisms (Avraham et al., 2015). Similarly, Saliba et al. (2017) demonstrated by single–cell RNA-seq analysis that heterogeneity of bacterial growth rate in individual macrophages could be correlated with different macrophage gene expression profiles.

Similar variability in both pathogens and macrophages could give rise to marked heterogeneity in susceptibility or resistance to TB encounters at body surfaces such as lung, skin and gut, in serosal cavities e.g., the pleura, pericardium and peritoneum (Rosas et al., 2014; Okabe and Medzhitov, 2016; Wang and Kubes, 2016), and within the blood or lymphatic circulation (Gordon et al., 2014b). At present, we know little about the impact of microbial heterogeneity and local and regional diversity of macrophages, on the initiation, dissemination and outcome of TB, well described in the early pathology literature. The mixed origin of MPS populations is further enhanced by mobilization from tissue reservoirs such as the spleen (Swirski et al., 2009) or the peritoneal cavity (Wang and Kubes, 2016), and by dynamic modulation of a spectrum of different cellular phenotypes, loosely described as “activation.” Organ-specific properties clearly relevant to pulmonary infection include the role of GM-CSF in alveolar macrophage development (Guilliams et al., 2013), their interactions with surfactant proteins and lipids (Hussell and Bell, 2014), as well as impact of the oxygen-rich environment and inhaled particulates. Moreover, the bone marrow, gut, liver and brain, for example, contain multiple subpopulations of resident macrophages, with different, but still largely undefined tissue-specific properties which will affect their response to MTB; these include differences in metabolism, microbicidal/microbistatic activity, persistence, latency and reactivation of infection. In addition, the presence of other micro-organisms, whether commensals or pathogens, can have a profound impact on macrophage-mycobacterial interactions. This is well illustrated in HIV/AIDS, a major co-pathogen of Tuberculosis, but subtler interactions with organisms in the microbiome of the gut, skin and lung, for example, and with antibiotics need further study. A particular illustration of genomic diversity of MTB as a result of spread within the lung as well as to extra-pulmonary tissues was provided by analysis of HIV-associated Tuberculosis at autopsy in subjects who had received minimal antitubercular treatment (Lieberman et al., 2016). Selection in individual patients resulted in co-existence of substrains for long periods, giving rise to repeated dissemination within, rather than new infections between individuals. It is not known whether this can also occur in the absence of AIDS.

In the present review we focus on macrophage properties relevant to the pathogenesis of tuberculosis during both innate and adaptive immunity to infection; further references can be found in recent multi-author volumes published by the American Society of Microbiology (Gordon, 2017; Jacobs et al., 2018); individual chapters are available in Microbiology Spectrum. We do not cover the role of polymorphonuclear leukocytes(PMN) and Dendritic cells(DC), except in passing. Box 1 summarizes the role of T lymphocytes in granuloma formation and macrophage effector functions. The potential roles of B lymphocytes and humoral responses including antibodies are beyond the scope of this review.

Box 1 **Adaptive immunity.**

The adaptive immune response, mainly comprised of antigen specific CD4+ cells, plays a critical role in the outcome of M. tuberculosis infection (Jasenosky et al., 2015). For robust control of the infection, T cells need to arrive timeously at the right site where they can interact with heterogeneous subpopulations of DCs, derived from circulating monocyte precursors and mature monocytes in blood (Austyn, 2016; Villani et al., 2017). Monocytes, essential effector cells in adaptive as well as innate immunity, are themselves heterogeneous and contribute to granuloma supply and demand at the core of the necrotic granuloma (Randolph, 2015; Pagan and Ramakrishnan, 2018). Apoptosis facilitates antigen presentation to T lymphocytes through MHC-I and CD1 on antigen presenting cells (APC) (Schaible et al., 2003). Typically, MTB- specific T cells become detectable at least 3 weeks after infection. In many TB models, establishment of the T cell immune response coincides with the arrest of bacterial replication (Flynn, 2006), indicating a significant role of adaptive immune T cell dependent—macrophage interactions in TB control. Two CD4+ T cell phenotypes essential for MTB control are Th1 and Th17 responses. Th1 immunity is characterized by the release of IFN-γ that activates macrophage microbicidal activities. IFN-γ is essential for anti-mycobacterial defense in mice (Flynn et al., 1993), and polymorphisms in the IFNG gene are associated with MTB infection risk and disease progression in humans (Rossouw et al., 2003). Th17 differentiation is driven by IL-23, and is characterized by the release of IL-17, which is critical for early recruitment of neutrophils to the site of infection (Freches et al., 2013). IL-17 has been shown to be required for protection against TB in mice infected with a hyper-virulent clinical isolate *M. tuberculosis* HN878 strain. Mice deficient in this cytokine had significantly higher bacillary burdens and less organized granulomas (Gopal et al., 2014). At excessive levels, however, IL-17 promotes tissue pathology and inflammation, suggesting that tight regulation of this cytokine is required during infection (Das and Khader, 2017). Foxp3+ T regulatory cells (Tregs) dampen Th1 immunity through inhibition of inflammatory cytokines by IL-10. Treg frequencies are higher in peripheral blood and at disease sites of patients with active TB, where they suppress production of MTB-specific IFNγ by Th1 cells *ex vivo* (Marin et al., 2010); too much expansion of these cells may result in impaired immunity.

The role of Th2 responses in TB infection is controversial. Mice deficient in IL-4, IL-4Rα, or STAT6 on a C57BL/6 background display disease outcomes similar to wild-type mice infected with MTB (North, 1998; Flynn and Chan, 2001; Jung et al., 2002). However, IL-4 seems to be detrimental in a BALB/c mouse background, with its deletion resulting in more protection against TB infections. Also, antibody neutralization of IL-4 on this background provides protection against chronic TB infection. Mechanisms associated with the detrimental role of Th2 cytokines remain unclear, but may stem from their ability to inhibit Th1 factors such as TNF, required to control bacterial replication (Hernandez-Pando et al., 2004). IL-4 is expressed in human surgically-derived lung tissues (Stanton et al., 2003), although its function in humans has not been established. Over-expression of another Th2 cytokine, IL-13, in mice, results in susceptibility to TB infection characterized by enhanced collagen deposition and enhanced lung pathology with necrotizing granulomas (Heitmann et al., 2014). These studies suggest that the IL-4/IL-13 axis drives pathological damage. A recent report by Minutti et al. (2017) has identified SP-A in the lung as a tissue-specific amplifier of IL-4-dependent macrophage proliferation and activation.

In granulomas, T cells constitute a larger part of the outermost layer. However, it is poorly understood how CD4+ T cells or IFNgamma penetrate lung lesions to access macrophage-rich areas at the centers. The Kaufmann laboratory has shown that chronic infection in both mice and humans is associated with the development of lymphoid follicle-like structures at the periphery of granulomas to orchestrate local host defense in the lung (Ulrichs and Kaufmann, 2006; Kahnert et al., 2007). They identified two layers surrounding the necrotic centers; the first, an inner layer that comprised CD68+ APCs and large cells, including epithelioid and Langhans giant cells. This layer also contained the bacilli, and CD4+ and CD45RO+ memory cells. The second layer was the outer area of the lesions, characterized by infiltration of B and T cells. This outer-most layer was enriched with CD8+ T cells, largely absent in the inner layer (Ulrichs and Kaufmann, 2006). Thus CD4+ and CD8+ T cells seem to exhibit distinct spatial distribution in a granuloma structure.

The importance of CD8 T cells in the long-term control of infection has been demonstrated using several mouse strains deficient in CD8 T cells due to different gene deletions (Lin and Flynn, 2015), but little is known about colocalisation of CD8 T cells and macrophages in granulomas. Depletion of this subset of cells in TB-infected mice results in an increased bacterial burden (van Pinxteren et al., 2000). The requirement for CD8 T cells was also shown in macaque models of TB, with some research indicating that the depletion of CD8 T cells in BCG-vaccinated macaques results in compromised control of MTB infection (Chen et al., 2009). Although more research is required in humans, data from macaques support the importance of CD8 cells in TB (Lin and Flynn, 2015). Other T cell subsets involved in immune responses to MTB infections and potential interactions with macrophage subpopulations include γδ T, innate lymphoid cells, NK and CD1-restricted CD4/CD8 double negative T cells (Jasenosky et al., 2015; Ndlovu and Marakalala, 2016).

The role of B cells and antibodies in TB remains largely unclear (Achkar et al., 2015); antibodies mediate potent effector mechanisms through macrophage Fc and Complement receptors and activation of their microbicidal mechanisms.

## MTB tropism for macrophages, entry and responses

The microbiology of MTB is under intense study, with emphasis on its metabolism, growth and unique constituents, such as complex surface glycolipids (Jackson, 2014), known to influence cellular infection. Although to a great extent tropic for macrophages and DC, it is clear that other cell types such as adipocytes are also infectible by MTB, as discussed below. Neutrophils can and do take up MTB, whether opsonized or not, and may die by pyroptosis as well as apoptosis. Human neutrophils which undergo MTB-induced necrosis can be ingested by macrophages (Dallenga et al., 2017) and MTB also replicates within necrotic human macrophages (Lerner et al., 2017, 2018). Recent studies have reported that activated macrophages can generate extracellular traps (METOSIS), comparable to, but distinct from NETOSIS (Doster et al., 2017).

Although there have been many attempts to identify the macrophage plasma membrane receptors responsible for MTB tropism, we still lack a clear understanding of the role of particular receptors and their heterogeneous expression by different tissue macrophage populations. This can be due to receptor redundancy, the low affinity of individual receptors, variable expression by different macrophages, *in vivo*, including species differences, and the presence of opsonins, rather than direct receptor-mediated uptake. The lectin-like mannose receptor (CD206) recognizes ManLam on MTB; its role in MTB uptake has been studied in considerable detail by Schlesinger and his colleagues (Kang et al., 2005). Other lectins that play a possible role in MTB infection of macrophages (Wilson et al., 2015; Rajaram et al., 2017) including Mincle (Ishikawa et al., 2009) and MCL; Beta2 integrins (CD11b/CD18) and Scavenger receptors such as MARCO and CD36, have also been implicated (Dodd et al., 2016). Siglecs (Crocker et al., 2007) expressed by a variety of immune cells, including macrophages, are sialic acid recognition sensors; desialylation of complex glycoconjugates can expose galactosyl residues, ligands for galectins (Sundblad et al., 2017). Identification of the contribution of particular receptors is complicated by the role of many of these macrophage surface receptors in clearance of apoptotic and necrotic cell targets, which are abundant in MTB infection and may even serve as potential Trojan horses for infection. These include Phosphatidyl Serine (PS) recognition molecules MerTK, axl (prominent in alveolar macrophages), their opsonic cofactors such as Gas and protein S, as well as other opsonins such as lactadherin (MFGE8), annexin, calreticulin, SP-A, and C1Q (Gordon and Plüddemann, 2018). Known MTB ligands for Mincle and MARCO include trehalose dimycolate (TDM, cord factor). Mincle together with MCL engages TDM to induce production of inflammatory cytokines such as IL-6, TNF, and IL-1beta (Ishikawa et al., 2009; Miyake et al., 2013). Dectin-2, DC-SIGN, and Mannose Receptor all recognize mannose-capped lipoarabinomannan (ManLAM) on the surface of MTB (Yonekawa et al., 2014; Marakalala and Ndlovu, 2017). Dectin-1, a major receptor of fungal beta-glucans that signals through the adaptor molecule CARD9, which is essential for tuberculosis control in the mouse (Dorhoi et al., 2010), has also been demonstrated to recognize an unknown ligand on the surface of MTB. This may differ from the well characterized beta-glucan ligand for Dectin-1 on fungi; such a ligand has been detected in selected tumor cells, and may be masked by N-glycans (Chiba et al., 2014).

Macrophage receptor-TB interactions result in activation of numerous cellular responses (Marakalala and Ndlovu, 2017). These molecules, however, are largely redundant in the *in vivo* control of chronic TB infection in mice and nor is it known which receptors are responsible for MTB uptake by the different macrophage subpopulations in granulomas, in situ, and for their resultant responses. For example, Dectin-1 knockout mice mount the same immune response as wild-type mice (Marakalala et al., 2011), whereas Dectin-2 has not yet been studied in an MTB *in vivo* model. MCL seems to drive a moderate protective role, while contradictory results have been reported for Mincle and DC-SIGN (Marakalala and Ndlovu, 2017). Future research should explore potential cooperation (Bezbradica et al., 2014) between individual CLRs, their synergy with other receptors such as TLRs and potential scaffold proteins in lipid rafts, and how this might affect the outcome of TB disease *in vivo*. Studies in human populations should confirm or identify novel dominant or recessive genetic susceptibility to MTB infection and BCG.

Macrophages express a range of plasma membrane and endosomal receptors for uptake of MTB components; exchange of mycobacterial and host-derived lipids is considered further below. Contrasting results have been reported on the role of TLRs in TB. TLR-2 and TLR-4, for example, have been shown to play a role in the long-term control of *M. tuberculosis* (Jo, 2008). Some studies, however, have reported that TLR2/4/9–/– triple knockout mice mount the same T cell mediated immune response compared with WT mice after infection with MTB (Holscher et al., 2008). Macrophages and other myeloid leukocytes express activatory and inhibitory FcR for IgG, as well as CR3 and other regulatory receptors for complement and antibody-coated immune complexes; these play a potentially important role in their endocytic and cytotoxic responses. Cytosolic sensors of MTB constituents and nucleic acids have been shown to play an important role in macrophage responses to MTB infection. For example, the nucleotide-binding oligomerization domain protein NOD2 has been shown to synergize with TLR-2 for the production of pro-inflammatory cytokines in response to live MTB (Jo, 2008). Another widely studied cytosolic sensor is the NLRP3 inflammasome, which induces IL-1beta processing and pyroptosis through caspase-1. Mishra et al have linked the NLRP3 inflammasome with lung pathology in TB infection, which can be regulated by NO (Mishra et al., 2013). Another report, however, has demonstrated that the inflammasome is not linked to the disease outcome (Walter et al., 2010). The recognition of MTB DNA will be described below after first considering the membrane dynamics and fate of the MTB phagosome in macrophages.

D'Arcy Hart and colleagues (Goren et al., 1976) first reported that MTB inhibited phagosome-lysosome fusion and acidification within the vacuolar compartment in which they resided after uptake, an important evasion mechanism for this intracellular pathogen. The question of entry to the cytosol by mycobacterial constituents and potential antigens for CD8 cytotoxic T lymphocytes has been under considerable debate for some years. It is likely that macrophage activation by CD4 T lymphocyte- and NK–dependent Interferon gamma overcomes both the fusion and acidification block. Macrophages are not only active endocytic and phagocytic cells, but also highly regulated secretory cells (Nathan, 2012), characterized by extensive membrane flow, selective intracellular fusion of vesicular membranes, fission and recycling (Tan and Russell, 2015). It is not known in detail how membrane traffic is selectively altered by MTB, and the impact of autocrine and paracrine effects of cytokines such as TNFalpha must also be kept in mind (Caldwell et al., 2014). Small GTPases play an important role in regulating intracellular survival of Mycobacteria in mouse macrophages; there are significant species differences in this regard (Gazzinelli et al., 2014). Another, genetic, difference in mouse susceptibility to infection relates to N-Ramp1 (Natural resistance-associated macrophage protein,1), a member of a metal ion transporter family at the interface between the phagolysosomal compartment and the cytosol (Buschman and Skamene, 2001; Blackwell et al., 2003). Recent studies with other Gram negative and Gram positive bacteria have described a role for a family of Copper-binding molecules (Burstein et al., 2005; Phillips-Krawczak et al., 2015) which participate in cytosolic multi-protein complexes and interact with the phagocytic pathway, lysosomal biogenesis and inflammasome activation. Their role in MTB infection has not been defined. Russell (Tan and Russell, 2015), Grinstein (Fairn et al., 2009), and their colleagues have made important contributions to studying acidification mechanisms in macrophages, and their role in anti-TB drug development, which requires screening within living macrophages and not in isolation. The interaction of MTB and the autophagy pathway (Deretic and Klionsky, 2018) has also attracted considerable attention, bearing on macrophage death and its role in immune activation and pathogenesis, as well as on mycobacterial survival and latency. Live imaging of spatiotemporal dynamics of MTB phagosomes has revealed that effective control of MTB replication in IFN-γ activated macrophages requires appropriate spaciousness of proteolytic phagosomes whose generation and membrane integrity are maintained by a Rab20-dependent membrane traffic pathway (Schnettger et al., 2017). Further studies on the proteome of isolated phagosomes from infected and uninfected cells as well as super resolution and intravital microscopy will help to clarify the complex interplay between the macrophage and its infectious cargo (Mahamed et al., 2017; Schnettger et al., 2017).

Intracellular bacteria, viruses and parasites have evolved a variety of different strategies to survive within macrophages; TB provides a particularly challenging infection model of great interest to cell biologists, microbiologists and immunologists. Although there has been an explosion of knowledge bearing on cytosolic sensing and intracellular signaling mechanisms of bacterial membrane molecules (NLRs) and nucleic acids (RIG-I, STING), the recognition of mycobacterial constituents by these intracytoplasmic protein complexes remains largely unknown. Direct detection of cytosolic MTB DNA by cGAS has been demonstrated by Watson et al. (2015). Unlike Listeria monocytogenes which produces the STING agonist cyclic-di-AMP, cGAS (cyclic GMP-AMP synthase) is required to activate type1 interferon by cytosolic DNA of MTB and Legionella pneumophila, via the STING/TBK/IRF3 pathway. Human genetic auto-inflammatory syndromes and mouse genetic models should have much to teach us in this regard.

## Granuloma formation in the lung

Alveolar macrophage infection is an important and well-studied initiator of granuloma formation. As noted above, they are exposed via the airway to MTB, which they capture and sense through plasma membrane, endosomal and cytosolic receptors. They generate antigens and interact with DC, which are required to activate naïve T lymphocytes, although macrophages can activate primed T cells (Austyn, 2016). Alveolar macrophages communicate with local epithelial cells to maintain gas exchange and set the threshold and the appropriate quality of the immune response. Macrophage populations in the lung are heterogeneous in their initial embryonic and subsequent bone marrow origin; monocyte recruitment to the lung is enhanced by exposure to particulates and pollutants that reach the lower airways (Torrelles and Schlesinger, 2017). As described above and in Figure 1, Russell and colleagues have demonstrated that ontogeny contributes to the interaction and outcome of initial MTB infection in the mouse (Huang et al., 2018). The authors favor a pre-programmed model ascribed to developmental origin, rather than responses to different local microenvironments. The receptors that mediate acute infection in this *in vivo* model were not defined. Apart from exposure to local surfactant proteins, potential opsonins and high levels of oxygen in the microenvironment are other macrophage modifying factors. Surfactant proteins A and D contribute to innate immunity outside the lung as well as within the alveolar space and may also contribute to extra-pulmonary TB (Ujma et al., 2017). The lung contains other specialized populations such as interstitial macrophages, that are not directly exposed to exogenous alveolar stimuli; these include intra-epithelial macrophages and DCs, found mostly in larger airways, and macrophage populations in the pleural cavity (Gordon et al., 2014a; McClean and Tobin, 2016). Alveolar macrophages undergo tissue imprinting in the lung as a result of their unique environment and exposure to infectious agents via the airways (Hussell and Bell, 2014). Other cells that are involved in the early response to TB infection are PMNs, which are the first defensive cells to be recruited to tissue during infection. PMNs have been reported to be the most abundant infected cells in the airways, sputum and BAL from patients with active TB.

### Heterogeneity in TB granulomas

**Figure 2:**
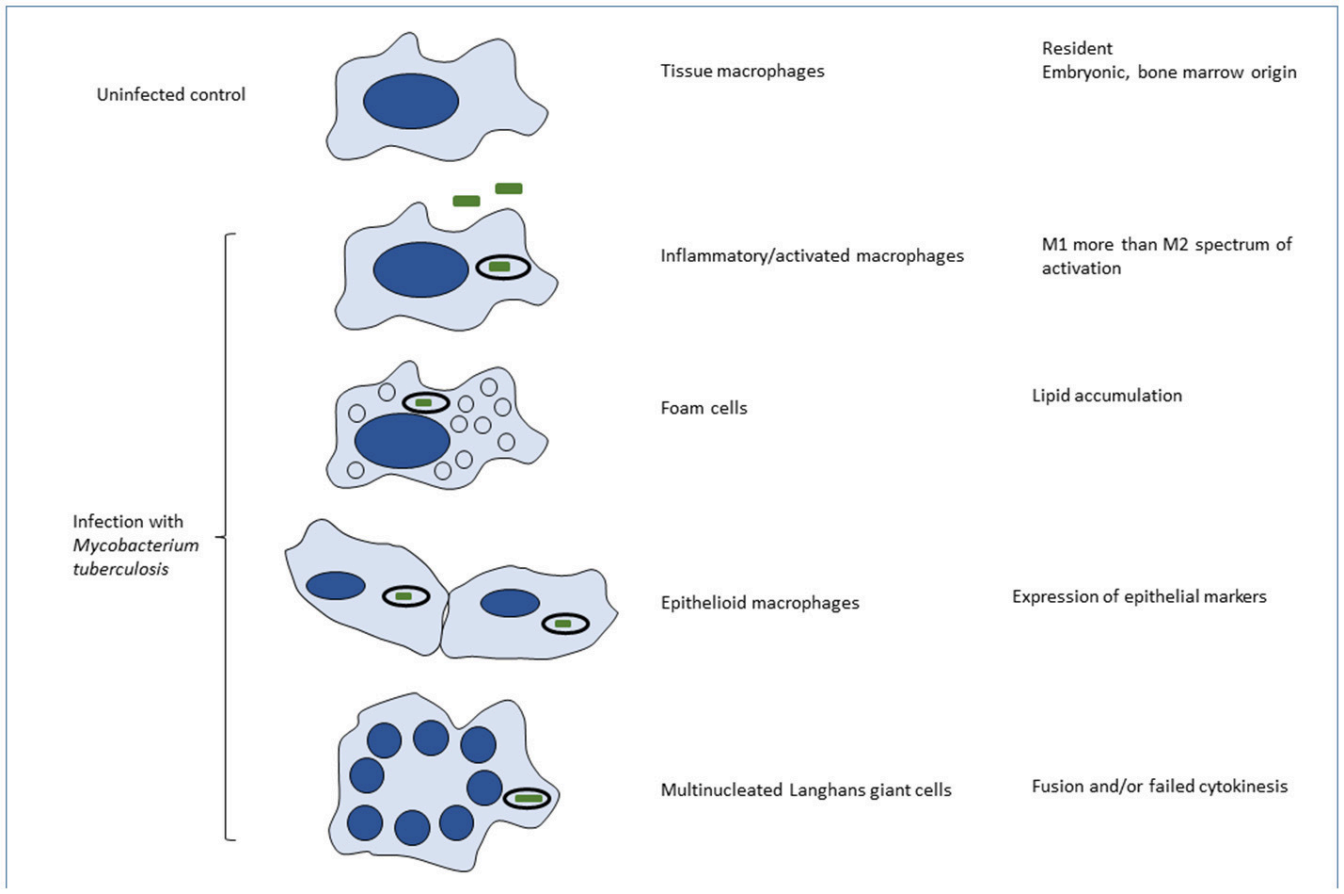
Phenotypic heterogeneity of macrophages induced by MTB infection *in vivo*. Macrophages are able to undergo remarkable variations in their phenotype *in vivo*, yet the mechanisms are poorly understood. This is partly the result of species variation, inadequacy of defined *in vitro* conditions to mimic *in vivo* phenotypes, limited population and single cell analysis of cells in situ or after extraction, *ex vivo*, and above all, the complexity and heterogeneity of the mixed populations present in tissues. Variables that have been characterized to some extent, include: ontogeny (embryonic or bone marrow origin); steady state and physiological conditions in different organs and microenvironments; sterile injury and acute or chronic infections such as MTB; cell recruitment, phagocytosis and activation by different exogenous and endogenous stimuli; and cell death by apoptosis, necrosis or pyroptosis. In many situations the macrophage phenotype covers a spectrum of responses, rather than binary alternatives. Their characterization is morphological, utilizing combinations of marker antigens, signatures of gene expression, and, where known, functional differences. The text provides examples of the different phenotypes illustrated here. The impact of macrophage phenotype on MTB infection, and conversely, of MTB infection on macrophage functions is barely understood.

The overall composition and fate of granulomas have been briefly described above, with emphasis on the heterogeneity of macrophage origin, anatomic location and phenotypes. Here we consider heterogeneity of MTB-induced granulomas in the lung. Recent data in human disease and primate models have shown that infected individuals contain a heterogeneous mixture of granulomas in various histologic states, with varying degree of immune activation, macrophage immune-phenotypes and the ability to control bacterial replication (Stanton et al., 2003; Kim et al., 2010; Lin et al., 2014; Gideon et al., 2015). Also, some important immune pathways seem to be distinctly enriched in specific regions within granulomas (Marakalala et al., 2016). This body of research suggests that disease-driving processes are compartmentalized within individual granulomas, and that these spatial processes may be linked to the clinical outcome. Our understanding of granuloma organization has come mostly from histopathology, which assesses structure by the abundance of various cell-types (Ulrichs et al., 2004; Gideon et al., 2015). Histological analyses of human TB have provided evidence for morphologic heterogeneity among pulmonary alveolar macrophages, foam cell formation, epithelioid cells and multinucleated giant cells (Figure 2; Dannenberg and Rook, 1994). Distinct phenotypes and activation status of macrophages in human pulmonary granulomas have also been demonstrated by immunohistochemistry with a panel of antibodies (Stanton et al., 2003). However, more research is still required for better understanding of macrophage differentiation, cell-to-cell interactions, heterogeneity and functions within various compartments of TB granulomas.

With the availability of new advanced techniques, research on the role of granulomas in TB has received considerable attention recently, with many advanced genome-wide approaches explored to give a better molecular characterization of the macrophage-rich structures. Using a combination of laser microdissection and microarray analysis of human lung tissues, a study by Kim et al. (2010) demonstrated that genes involved in lipid sequestration and metabolism were highly expressed in caseous granulomas. Immunohistochemical analysis of the tissue confirmed the upregulation of proteins involved in lipid metabolism in cells surrounding the caseum, including adipophilin, acyl-CoA synthetase long-chain family member 1 and saposin C.

In a similar approach, Marakalala et al. (2016) utilized proteomics and mass spectrometric imaging to characterize spatial organization of inflammatory pathways within human granulomas. The centers of the granulomas were concentrated with pro-inflammatory and antibacterial signatures whereas the cellular peripheries were enriched with anti-inflammatory, tissue-preserving mediators (Marakalala et al., 2016). A similar phenomenon has been shown in granulomas from macaques, in which spatial delineation and inflammatory cell programmes were organized around distinct subsets of macrophages (Mattila et al., 2013). These studies suggest that local balance of pro- and anti-inflammatory responses may determine the fate of individual granulomas, which collectively influence the host response (Marakalala et al., 2016). The model proposed by these studies may also help to explain the heterogeneity that is often observed in TB granulomas, even within the same host, as differences in inflammatory responses probably contribute to diverse granuloma structures and functions (Cadena et al., 2017). In macaques, Lin et al demonstrated that the majority of granulomas begin with a single bacterium (Lin et al., 2014). The authors observed variability in bacterial killing within individual granulomas that was independent of host status. These findings established that local events at granuloma level are likely to influence the clinical outcome of infection. To understand the repertoire of the adaptive immune system in various granulomas, Subbian et al. examined lung granulomas from patients with chronic pulmonary TB (Subbian et al., 2015). The study revealed significant variability in T cell density and fibrosis. Gideon et al reported variability in different factors even within the same macaque (Gideon et al., 2015). These factors included the total numbers and phenotypes of T cells, the numbers and heterogeneity of macrophages and a wide range of cytokine profiles and bacterial burdens within each granuloma. The majority of these T cells produced only a single cytokine; dominant cytokines included IFNγ, interleukin-2 (IL-2), Tumor Necrosis Factor (TNF), IL-10, and IL-17. These findings suggest that granuloma heterogeneity and spatial organization of cellular and humoral host responses at granuloma level should be taken into account when designing host-directed therapies that limit lung pathology.

### Epithelioidisation of macrophages within granulomas

Macrophages can undergo a series of morphological changes characterized by cytoplasm expansion and interlocking between membranes of neighboring cells. Macrophages undergoing this histological transformation have always been termed epithelioid cells (Dannenberg and Rook, 1994), although their significance in granuloma functions remains unclear. Recent work by Cronan et al. has meticulously described molecular underpinnings of macrophage epithelialization within granulomas (Cronan et al., 2016). Using a zebrafish model, immunofluorescence and live imaging techniques, the authors showed that as macrophages create junctions with each other to form a tuberculous granuloma, they express and deploy adherens components. The macrophages expressed proteins that are typically associated with epithelial cells, including desmosomal proteins (desmoplakin, desmoglein, and desmocollin), adherens junction proteins (E-cadherin, plakoglobin, α-, β-, and δ- catenin), and tight junction pathways (ZO-1 and ZO-2). Proteins corresponding to these genes have been identified in a proteomic survey of granulomas from humans infected with MTB (Marakalala et al., 2016). A significant finding from the study by Cronan and colleagues is the observation of epithelial markers in the epithelioid cells of granulomas. Taken together, these studies underpin epithelioidisation of tissue macrophages as a fundamental process in granuloma development and the ability to control infection in zebrafish and humans.

## Macrophage activation: gene expression and phenotype modulation

BCG has provided a potent adjuvant to study for its own sake as a vaccine, as well as providing a tool to induce innate and adaptive immune activation of macrophages. Mackaness and colleagues performed now-classic studies on the role of BCG administration in mice, in order to induce enhanced resistance, not only to BCG, but also non-specifically to an unrelated challenge, e.g., Listeria monocytogenes, and vice versa (Carter, 2014). Building on earlier pioneering discoveries by Koch and Ehrlich, as well as Metchnikoff, the Mackaness group confirmed that this mechanism, now termed “Classical macrophage activation,” was cellular and not humoral in origin. Subsequent studies identified Interferon gamma as the major cytokine produced by specific CD4 T lymphocytes or by innate NK cells. The role of IL-12 and IL-18, and various cytokine receptors on macrophages was elucidated by several investigators; these studies complemented the discovery by Casanova (Casanova and Abel, 2017), Holland (Wu and Holland, 2016) and others of primary immunodeficient patients unable to control BCG infection after immunization with live BCG or after infection by relatively avirulent organisms. Studies by Nathan (Nathan, 2013) and others established that the induction of inducible nitric oxide synthase (i-NOS) and generation of antimicrobial nitrogen-based radicals, combined with oxygen radicals generated by the respiratory burst, contributed to anti-microbial resistance in mice. After some technical issues, the role of NO in human infection by MTB was confirmed. Subsequent microarray analysis by several research groups revealed a signature of gene expression in mouse and humans after IFN gamma activation of macrophages, distinct from the “alternative activation” signature induced by the Th2 cytokines IL-4 and IL-13, acting through the common IL-4R alpha chain receptor. Nathan's group also documented potent metabolic anti-oxidant defenses in macrophages. Vitamin D induces antimicrobial peptides and autophagy in monocyte/macrophages and promotes resolution of inflammation in TB patients (Fabri et al., 2011; Coussens et al., 2012). Glucocorticosteroids have potent inhibitory effects on inflammation, monocyte recruitment and activation of macrophages by MTB.

There is evidence that TB infection in humans induces IL-4 gene expression in macrophages (Stanton et al., 2003), possibly mediated by TLR involvement, the spectrum of gene activation that is upregulated indicates a more mixed activation signature than in laboratory mouse strains. The initial binary classification of M1- and M2-types of macrophage activation by IFN gamma and IL4/13 respectively has been refined to a broader spectrum of activation, in which individual markers are replaced by a signature of a group of genes, many of which overlap with altered gene expression by other defined stimuli. Alternative activation of macrophages has been implicated in tissue repair and fibrosis; whether this plays a role, together with transforming growth factor beta, in Tuberculosis-induced fibrosis remains to be elucidated. Although Th2 cytokines readily induce macrophage cell-cell fusion *in vitro* and *in vivo*, the mechanism of Langhans giant cell formation (Figure 2), a characteristic feature of human tuberculosis, remains obscure. Interferon gamma by itself does not promote fusion *in vitro*, but may contribute to macrophage fusion in combination with ligation of uncharacterized macrophage plasma membrane glycoproteins (Sakai et al., 2012), GM-CSF and fusogenic MTB (Puissegur et al., 2007) and host lipids. An alternative mechanism, DNA injury and failure of cytokinesis has been proposed to account for multinuclear giant cell (MNGC) formation in tuberculosis (Herrtwich et al., 2016). The functional significance of Langhans giant cell formation, especially in relation to interactions with MTB, remains unclear, Studies on IL-4 induced MNGC have revealed enhanced clearance of large particulates and systemic amyloid deposits *in vivo*; this was associated with selective activation of CR3 function, as a result of cell fusion (Milde et al., 2015). Recent revival of interest in macrophage metabolism has provided evidence that classically and alternatively activated macrophages utilize glycolysis and oxidative phosphorylation, respectively (O'Neill and Pearce, 2016), as their main source of energy. Shi et al have reported that infection with MTB induces the Warburg effect in mouse lungs (Shi et al., 2015). Gleason, O'Neill and Keane have shown that enhanced aerobic glycolysis in human alveolar macrophages is required to control intracellular bacillary replication (Gleeson et al., 2016).

As an adjuvant, BCG has been utilized to boost anti-cancer immunity, with some success in bladder cancer after local administration (Alexandroff et al., 1999). This could benefit combination therapy with antibody-based checkpoint therapy targeting T lymphocyte co-stimulatory receptors. Extending earlier findings that BCG could prime macrophage activation, Netea and colleagues (Arts et al., 2018) have used BCG as one of their protocols to induce “trained immunity,” ascribed to epigenetic mechanisms. Yeast particles, taken up by macrophages through the beta-glucan receptor, Dectin-1, which signals through syk and Card9, provides a similar priming function. As information accumulates from GWAS and SNP studies, other genes than those mentioned above and the MHC complex may be found to contribute to human macrophage activation.

## Protein secretion by activated macrophages: role in pathogenesis of tuberculosis

It is only since the early 70's, from the description of lysozyme secretion by mouse macrophages in cell culture (Gordon et al., 1974), that it was recognized that macrophages are not only professional phagocytes, but also highly active secretory cells. Mature macrophages do not accumulate preformed proteins in granules as do granulocytes, for immediate release in acute inflammation. Low molecular metabolites derived from oxygen during the respiratory burst and from preformed lipids during acute inflammation can be rapidly generated from arachidonates in the plasma membrane, but proteins are newly synthesized, consistent with their required functions in prolonged inflammation, as well as in tissue homeostasis. Macrophages produce a large variety of proteins in relatively small amounts, e.g., all components of the complement cascade, potentially significant in their immediate local environment. Lysozyme is constitutively released by macrophages in culture, although its production is greatly enhanced by chronic inflammation and activation, as seen by in situ hybridization in BCG granulomas (Chung et al., 1988). It is highly cationic, will bind to host negatively charged proteoglycans, but enzymatic activity seems restricted to bacterial wall rather than host substrates, directly or after exposure, eg through complement lysis, as in MTB. It is lost from plasma by filtration through the kidney (MW 14 Kd), but can be elevated by increased numbers of macrophages, as well as enhanced output per cell, as in tuberculosis.

Activated macrophages produce neutral proteinases such as urokinase, elastase, collagenases and other metalloproteinases, implicated in fibrinolysis, tissue remodeling and degradation. Caspases are released as a result of inflammasome activation; recent studies have demonstrated a range of caspase functions unrelated to cell death (Shrestha and Megeney, 2012). As part of inflammatory regulation, macrophages also release alpha 1 antitrypsin and alpha 2 macroglobulin to inhibit proteolytic activity. More restricted cleavage can activate proenzymes, e.g., Angiotensin-converting enzyme (ACE) (Bernstein et al., 2013), or inactivate components of plasma protein cascades, e.g., by carboxypeptidase action, a macrophage-specific enzyme (Mahoney et al., 2001). The protein cross-linking enzyme Transglutaminase 2, a conserved feature of alternative activation of macrophages (Martinez et al., 2013), has been implicated in MTB restriction by macrophages (Palucci et al., 2017), as well as in fibrosis. FXIIIA, a distinct transglutaminase, also expressed by macrophages, displays similar properties (McGovern et al., 2014). Macrophages can release a large number of proinflammatory (e.g., Type I interferon, TNF alpha, Interleukin 1 beta, IL-12, IL-18, IL-6) and anti-inflammatory cytokines (IL-10 and TGFbeta), and a range of CC, CXC and CX3C chemokines, as well as expressing their receptors. Growth factors which macrophages produce, and respond to, include M-CSF, GM-CSF and Erythropoietin, as well as releasing vascular endothelial and fibroblast growth factors.

Release of TNF alpha from the plasma membrane is mediated by ADAM 17, a metalloproteinase; surface TNF is an essential ingredient of granuloma formation (Fremond et al., 2005) and may play a role in contact-dependent killing of viable, TB- infected macrophages (Mahamed et al., 2017), whereas secreted TNF may be important in catabolism and weight loss, with IL-6 (Flint et al., 2016). Recent findings by McIlwain et al (McIlwain et al., 2012) and the Freeman group (Christova et al., 2013) have shown that a Rhomboid family of multispan transmembrane molecules, of which i-Rhom 2 is specific for macrophages, are essential for Adam17- dependent release of TNF alpha from the macrophage plasma membrane, and may also contribute to chaperone activity in ER transport and in lysosomal stability. Taken together, it is clear that macrophage secretory activity plays a major role in granuloma formation and function. To establish which macrophage genes are expressed at the single cell level, both as message and protein, it will be necessary to examine samples of TB-infected tissue *in situ* and *ex vivo*. It will also be necessary to relate macrophage activities within and among granulomas and to characterize heterogeneity of macrophage sources and functions of different products within lesions.

## Type I interferon and tuberculosis

Macrophages are an important source of Type 1 Interferon in inflammation. Studies by Berry et al. (2010) identified a whole blood transcriptional signature in active TB patient cohorts from the United Kingdom and South Africa. The signature was dominated by IFNα/β-inducible genes, which were overexpressed in neutrophils. It is likely that blood and activated tissue macrophages are a major source of type I IFN as well, since the type I IFN gene profile also correlated with the extent of radiographic disease. Interestingly, the whole blood IFN signature was diminished with successful TB treatment, suggesting an association of the interferons with disease severity. Additional studies have verified the potential detrimental role of type I IFNs in TB (McNab et al., 2015).

A recent study by Zak et al investigated blood RNA expression to predict progression from latent tuberculosis infection to active disease (Zak et al., 2016). In the study, latently infected South African adolescents were followed for 2 years. Blood samples from latent infections that converted to active disease were analyzed by RNA sequencing to identify genes that were differentially expressed in comparison to the controls who did not convert. A 16-gene signature was identified and validated using quantitative real-time PCR (Zak et al., 2016). Interestingly, the signature comprised interferon-inducible genes previously identified as expressed in active tuberculosis (Berry et al., 2010). This confirms that type I interferon signatures can be exploited to predict disease progression in point of care testing.

## Macrophage eicosanoids and tuberculosis

**Figure 3:**
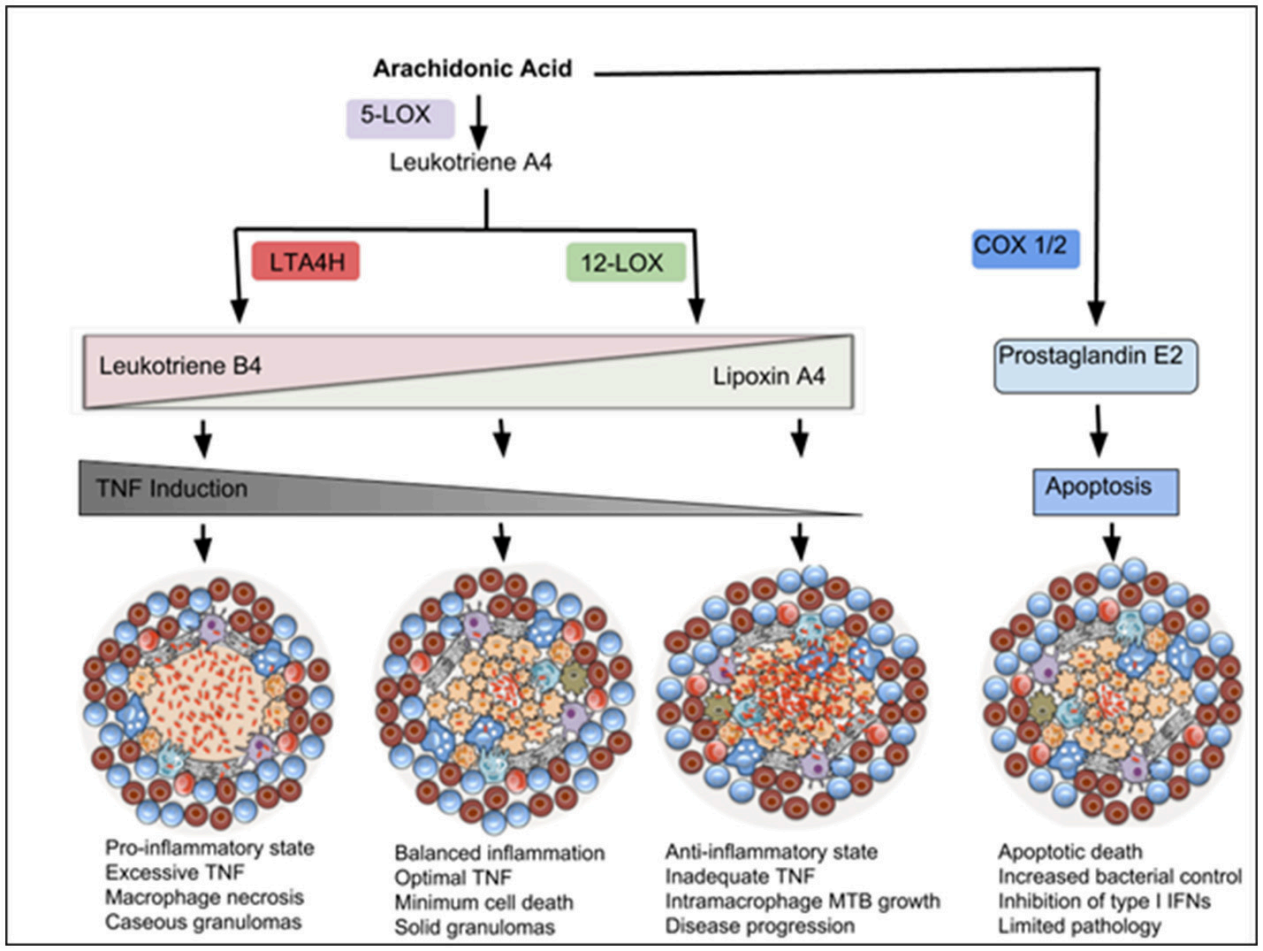
Eicosanoids drive inflammatory responses that modulate macrophage cell death and TB disease outcome. Arachidonic Acid (AA) can be metabolized by 5-LOX to produce Leukotriene A4 (LTA4), which is a precursor of Leukotriene B4 (produced via an enzyme LTA4H) or Lipoxin A4 (produced via 12-LOX). Leukotriene B4 (LTB4) induces pro-inflammatory responses and TNF expression, which in excess, promotes macrophage necrosis and granuloma caseation. Lipoxin A4 (LXA4) is anti-inflammatory and associated with TNF deficiency and increased intra-macrophage bacterial growth. Balanced amounts of these pro- and anti-inflammatory lipid mediators are required for optimal control of cell death and disease progression. AA can also be metabolized by COX-1/2 to produce Prostaglandin E2, which induces macrophage apoptosis and promotes antimicrobial activities.

Macrophages are a major source of Arachidonate metabolites which have been shown to be important regulators of inflammation and the outcome of tuberculosis and granuloma formation (Figure 3). Although the mechanisms driving type I IFN-mediated disease progression are still poorly understood, a few studies have suggested suppression of pro-inflammatory cytokines and Th-1 immunity as a major factor. A study by Mayer-Barber and colleagues demonstrated a detrimental role for type I IFNs in TB infection in mice (Mayer-Barber et al., 2014); inhibition of IFNalpha/beta reversed the detrimental effects of type I IFNs. This response was dependent on the induction of prostaglandin E2 (PGE2). Direct administration of this prostanoid resulted in the control of bacterial infection through inhibition of type I IFNs (Mayer-Barber et al., 2014). This study indicated the importance of type I IFNs and manipulation of the eicosanoid balance as a target for host- directed therapies.

Studies in mice and zebrafish models have linked the eicosanoid pathways with various modes of macrophage death. Virulent MTB H37Rv has been shown to drive macrophage necrosis through the production of lipoxin A4 (LXA4), which inhibits prostanoid synthesis and promotes mitochondrial inner membrane perturbations. In contrast, prostaglandin E2 (PGE2) suppressed inner mitochondrial membrane damage and inhibited macrophage necrosis in macrophages. Furthermore, mice deficient in prostaglandin E2 synthase (PGES) had higher lung bacillary loads compared with wild type mice, indicating the protective role of prostaglandin E2 against mycobacterial infections in the lung (Chen et al., 2008). ALOX5 knockout mice, which cannot produce LXA4, undergo more apoptosis with enhanced MTB-specific T cell responses (Divangahi et al., 2010), and are more resistant to MTB infection (Bafica et al., 2005), suggesting detrimental effects of LXA4 during infection. On the other hand, mice deficient in COX-2 are more susceptible to MTB infection, confirming an important role for prostanoids (Mayer-Barber et al., 2014).

Using a zebrafish model, Tobin and co-workers studied the contribution of eicosanoid mediators in response to *Mycobacterium marinum* (Mm) infection (Tobin et al., 2010, 2012). The authors identified a critical role for the enzyme Leukotriene A4 Hydrolase (LTA4H) in determining susceptibility of zebrafish to infection. LTA4H is responsible for production of Leukotriene B4, a pro-inflammatory eicosanoid that induces the transcription of TNF. Excess LTA4H in Mm-infected zebrafish led to early reduction of bacterial growth inside macrophages. However, bacterial restriction was rapidly followed by macrophage necrosis, which enabled bacterial replication in the permissive extracellular milieu (Tobin et al., 2010, 2012). These results suggest that LTA4H, through TNF production, can dually mediate resistance and susceptibility to mycobacterial infection (Roca and Ramakrishnan, 2013). Its absence leads to TNF deficiency, resulting in increased bacterial growth intracellularly, while its overexpression leads to excessive production of TNF resulting in macrophage necrosis (Figure 3; Tobin et al., 2012; Roca and Ramakrishnan, 2013). In human tuberculous granulomas, LTA4H is abundant in necrotic regions, where it colocalises with TNF (Marakalala et al., 2016). In humans, the genetic status of LTA4H has been shown to be critical for disease outcome in TB meningitis patients. Patients who are homozygous for low or high LTA4H expression alleles developed severe TB meningitis with reduced survival, while those heterozygous for intermediate LTA4H expression controlled the infection (Tobin et al., 2012). These findings confirm a need for balance in inflammatory responses, much of it orchestrated by metabolic changes in activated macrophages.

## Macrophage foam cell formation and caseation

Foam cell formation and caseation are hallmarks of human tuberculosis, yet their genesis, lipid composition, and significance for outcome remain poorly understood. Caseation could be due to cell death, resulting from mycobacterial toxicity and/or an exuberant host immune response, as well as altered lipid metabolism. Possible mechanisms include mobilization from lipid stores, endocytosis and deposition in macrophages, enhanced synthesis, locally and systemically, and/or failure to degrade accumulated lipids in macrophages. Macrophage foam cells form readily after uptake of various protein-bound lipids by a range of scavenger receptors, e.g., SR-A, CD36 and LDL receptors (Neyen and Gordon, 2014). Lipid droplets arise by synthesis and export from the endoplasmic reticulum to the cytosol, before becoming bound by vesicle membranes (Figure 2). Macrophage synthesis, uptake and exchange of lipids are regulated by transcription factors such as PPAR gamma and cytokines released by T lymphocytes and adipocytes (Daniel et al., 2011; Ouimet et al., 2016; Cambier et al., 2017). Foam cells in mouse granuloma models have been shown to express Wnt 6, involved in macrophage arginase1 induction and proliferation (Schaale et al., 2013). In addition to MTB-macrophage interactions, there seems to be an axis of interactions among MTB, macrophages and adipocytes, eg multinucleated macrophages are closely associated with adipocytes in white adipose tissue crown-like structures (Touton cells) and there is evidence that MTB can infect adipocytes (Neyrolles et al., 2006; Agarwal et al., 2016). Lipids derived from intracellular bacteria and/or plasma are potentially fusogenic, contributing in addition to host cytokines to Langhans giant cell formation. At a systemic level there is an association between TB and diabetes, cachexia is a characteristic feature of advanced TB, possibly mediated by TNF (originally named cachectin) and IL-6, and there is more than a superficial resemblance between caseation and atheroma, a hallmark of non-infective atherosclerosis. Recent studies have drawn attention to cholesterol (Huang et al., 2018) and oxysterol metabolism (Lu et al., 2017) in immune cells and of phenolic glycolipids in experimental mycobacterial infection (Cambier et al., 2017). Huang et al. have stressed the capacity of MTB to access and degrade fatty acids and sterols in order to survive within macrophages. To gain a better understanding of prolonged host-pathogen interactions in chronic TB, it will be necessary to perform lipidomic analysis of the heterogeneous macrophage populations as well as MTB, ideally, *in vivo*, and at the single cell level.

## Conclusions

Tuberculosis has been well documented as a clinical and pathological entity, yet many aspects of the disease remain unclear; while its rich natural history provides unique opportunities for fundamental and applied research. Since the nineteenth century it has been known that macrophages are an integral component of its pathogenesis and sequelae. In this review we have shown how cellular and molecular advances have generated new insights into the role of macrophages and, conversely how tuberculosis research continues to provide a wealth of theoretical and practical problems for study of macrophage immunobiology. Although we are beginning to understand the phenotypic distinction between tissue- resident yolk sac-derived and inflammatory bone marrow- derived macrophages in the mouse, this concept needs validation in humans; moreover, we do not know the impact of specific organ localization as well as origin on macrophage- MTB interactions. The extensive heterogeneity of macrophage populations *in vivo*, revealed by population and single cell analysis, mandates exquisite selectivity to target particular subsets of infected cells, without collateral injury to the host, as emphasized by Srivastava et al. (2014). Furthermore, the associated caseation and fibrosis, hallmark complications of the human disease, are poorly expressed in experimental models and *in vitro*; distinctions between the immune response to TB in humans and in animal models are emphasized by Scriba et al. (2017). Further progress will depend on access to human tissue from patients, the development of sensitive methods to study small amounts of tissue in situ, ideally by non-invasive methods over the course of the disease, and validation with *in vitro* models of human cells as well as development of new animal models that mimic the disease in humans. Shorter courses and more effective drug treatment of patients will be achieved by high throughput screening within macrophages to identify novel mycobacterial targets. The role of macrophages in granuloma and giant cell formation, epitheliodisation, caseation and fibrosis, all associated with TB, requires further investigation.

Study of tuberculous granulomas should promote understanding of other granulomatous diseases such as sarcoidosis and autoimmune destructive conditions such as Granulomatosis with polyangiitis, formerly called Wegener's granulomatosis. The co-epidemic of TB with HIV-1/AIDS, characteristically treated as a T cell failure, also involves macrophage dysfunction, as a viral reservoir, but especially when successful HAART restores CD4 T lymphocyte function, resulting in the Immune reconstitution inflammatory syndrome (IRIS) (Bell et al., 2017). Corticosteroid treatment, usually associated with increased risk in TB treatment, may be of benefit in preventing this outcome.

Apart from its remaining, but limited value as a vaccine, BCG offers a useful starting point to probe macrophage functions as an immunological adjuvant, in non-tuberculous intracellular infections and in selected cancer therapy. Recent benefits from BCG administration have been ascribed to imprinting haematopoietic stem cells in bone marrow to generate daughter macrophages with enhanced antimycobacterial effector function (Kaufmann et al., 2018). Dissection of the BCG genome should reveal new immunomodulatory prospects, without side effects resulting from unbridled macrophage activation. Perhaps most exciting will be to utilize such information in host-directed and combination therapy of drug-resistant Tuberculosis (Reiche et al., 2017), for example, targeting DNA replication and repair for the development of novel therapeutics against TB (Guler and Brombacher, 2015).

## Author contributions

SG conceived and wrote the manuscript. MM wrote the manuscript and produced the figure. FM wrote the manuscript. AP reviewed and revised the manuscript.

### Conflict of interest statement

The authors declare that the research was conducted in the absence of any commercial or financial relationships that could be construed as a potential conflict of interest.

## Footnotes

^1^WHO. World Health Organization Tuberculosis Fact sheet. http://www.who.int/mediacentre/factsheets/fs104/en/index.html. In, (January 2018).

## Abbreviations

Adam-17: disintegrin and metalloproteinase17
ADCC: antibody-dependent cellular cytotoxicity
ALOX-5: arachidonate 5-lipoxygenase
APC: Antigen presenting cell
BCG: Bacille Calmette Guerin
COX 1/2: cyclooxygenase 1/2
CTL: C-type lectin
DC: dendritic cell
DC-SIGN: dendritic cell-specific intercellular adhesion molecule-3-non integrin
FcR: Fc Receptor
HAART: Highly active antiretroviral therapy
HSC: haemopoietic stem cells
IRIS: immune reconstitution inflammatory syndrome
IgG: Immunoglobulin G
i-RHOM2: inactive rhomboid protein2
LTA4H: Leukotriene A4 hydrolase
LTB: leukotriene B
LXA4: lipoxin A4
ManLam: mannose-capped lipoarabinomannan
MARCO: macrophage receptor with collagenous domain
MCL: macrophage C-type lectin
Mincle: macrophage inducible Ca++-dependent lectin
Mm: mycobacterium marinum
MPS: mononuclear phagocyte system
MTB: mycobacterium tuberculosis
NLR: nod-like receptor
N-Ramp: natural resistance associated macrophage protein
PGE2: prostaglandin E2
PPAR: peroxisome proliferator activated receptor
SP-A: Surfactant protein-A
SR-A: Scavenger receptor A
STING: stimulator of interferon genes
TB: tuberculosis
TNF: tumor necrosis factor
TLR: Toll-like receptor
TRegs: regulatory T lymphocytes.
